# Comment on Giotta Lucifero et al. Impact of Irradiation on Post-Surgical Residuals of WHO Grade I Meningioma. *J. Clin. Med.* 2025, *14*, 5829

**DOI:** 10.3390/jcm14248790

**Published:** 2025-12-12

**Authors:** Eva María Corrales-García, Pedro David Delgado-López

**Affiliations:** 1 Radiation Oncology Department, Hospital Universitario de Burgos, Avda Islas Baleares 3, 09006 Burgos, Spain; evacorrales@yahoo.es; 2 Neurosurgery Department, Hospital Universitario de Burgos, Avda Islas Baleares 3, 09006 Burgos, Spain

We read with great interest the article by Giotta Lucifero and colleagues [1] published in the journal, analyzing long-term outcomes of patients with residual WHO grade I meningiomas treated with subtotal resection alone or in combination with radiotherapy. The authors conclude that irradiation of residual WHO I meningiomas was associated with increased recurrence, worse survival, less clinical control, and increased malignant progression.

While the large sample size and extended follow-up are commendable, we believe that several methodological limitations undermine the causal interpretation of the results. Patients allocated to radiotherapy were likely to harbor more complex tumors—often larger, recurrent, or located in surgically inaccessible regions—thereby introducing a substantial selection bias and confounding by indication that can independently explain the worse progression-free and overall survival observed in the irradiated group. Moreover, the study pooled heterogeneous irradiation modalities, including stereotactic radiosurgery, fractionated stereotactic radiotherapy, and proton beam therapy, delivered as either adjuvant or salvage treatment, with widely variable doses and fractionation schemes; such heterogeneity limits the validity of attributing uniformly negative outcomes to radiotherapy. Although Cox regression was applied, the absence of robust adjustment for key prognostic covariates such as tumor size, anatomical location, surgical accessibility, baseline performance status, and number of resections raises further concerns, as well as the lack of molecular data, increasingly relevant in the WHO 2021 and cIMPACT frameworks, makes it impossible to stratify patients according to biological risk. Additionally, the endpoint of “clinical control,” defined as the absence of subsequent interventions, is problematic, since irradiated patients are usually monitored with more intensive imaging protocols, introducing a surveillance bias that inflates recurrence detection and may not reflect true differences in tumor biology. Taken together, these issues make causal inferences precarious, the conclusion that radiotherapy itself increases recurrence, and malignant transformation should be tempered, since retrospective association does not establish causation.

However, it should be acknowledged that exposure to ionizing radiation represents the only confirmed environmental risk factor for the development of meningiomas and that radiotherapy may increase the mutational burden on irradiated tissues. Yet, this phenomenon is not unique to meningiomas but rather represents a universal biological consequence of radiotherapy across all tumor types. Importantly, this intrinsic mutagenic potential does not undermine the therapeutic value of radiotherapy, which remains a cornerstone of evidence-based management for selected patients with residual or recurrent WHO grade I meningiomas. Modern radiotherapy techniques—including stereotactic radiosurgery, fractionated stereotactic radiotherapy, and proton beam therapy—achieve durable local control with minimal toxicity when applied within appropriate clinical contexts [2,3,4].

In fact, there is substantial prospective and long-term evidence supporting the safety and efficacy of modern radiotherapy in selected patients with residual benign meningiomas. The prospective phase II NRG/RTOG 0539 trial demonstrated excellent outcomes in patients with residual low-risk WHO grade I meningiomas managed within a structured protocol, showing that long-term survival remains high and challenging the need to withhold radiotherapy indiscriminately [2]. Long-term radiosurgical series also report durable local control, with Lippitz and colleagues documenting approximately 88% tumor control at 10 years after Gamma Knife radiosurgery with acceptable toxicity [3]. Fractionated proton beam therapy has also shown excellent results, as reported by Holtzman and co-workers, who described a five-year actuarial progression rate of only 6% and severe toxicity below 2% in benign meningiomas treated with conformal proton therapy [4]. Furthermore, prospective phase II experiences with hypofractionated stereotactic radiotherapy for large or critically located meningiomas, such as the study by Alfredo et al., have demonstrated encouraging tumor control with manageable side effects [5]. Finally, international guidelines such as those of the European Association of Neuro-Oncology explicitly recommend considering radiotherapy in subtotally resected or recurrent WHO grade I meningiomas in surgically challenging locations, underlining that modern radiotherapy remains an integral and safe component of evidence-based management [6].

In light of these considerations, while the dataset presented by Giotta Lucifero et al. [1] is valuable for its length of follow-up, its methodological limitations and lack of robust adjustment for confounders significantly weaken the argument that irradiation per se worsens prognosis. The conclusions should therefore be interpreted with caution and framed as associative rather than causal, and current evidence continues to support a role for radiotherapy as a safe and effective treatment for selected patients with residual WHO grade I meningiomas.
